# Monolithic Use of Inert Gas for Highly Transparent and Conductive Indium Tin Oxide Thin Films

**DOI:** 10.3390/nano14070565

**Published:** 2024-03-24

**Authors:** Hessa I. Alabdan, Fahad M. Alsahli, Shubhranshu Bhandari, Tapas Mallick

**Affiliations:** 1 Environment and Sustainability Institute, University of Exeter, Penryn Campus, Cornwall TR10 9FE, UK; hima201@exeter.ac.uk (H.I.A.); fa419@exeter.ac.uk (F.M.A.); s.bhandari@exeter.ac.uk (S.B.); 2 Department of Physics and Renewable Energy, College of Science and Humanities-Jubail, Imam Abdulrahman Bin Faisal University, Jubail 35811, Saudi Arabia; 3 Physics Department, University of Hafr Al Batin, Al Jamiah, Hafar Al Batin 39524, Saudi Arabia

**Keywords:** indium tin oxide, thin films, RF magnetron sputtering, third generation solar cells

## Abstract

Due to its excellent electrical conductivity, high transparency in the visible spectrum, and exceptional chemical stability, indium tin oxide (ITO) has become a crucial material in the fields of optoelectronics and nanotechnology. This article provides a thorough analysis of growing ITO thin films with various thicknesses to study the impact of thickness on their electrical, optical, and physical properties for solar-cell applications. ITO was prepared through radio frequency (RF) magnetron sputtering using argon gas with no alteration in temperature or changes in substrate heating, followed with annealing in a tube furnace under inert conditions. An investigation of the influence of thickness on the optical, electrical, and physical properties of the films was conducted. We found that the best thickness for ITO thin films was 100 nm in terms of optical, electrical, and physical properties. To gain full comprehension of the impact on electrical properties, the different samples were characterized using a four-point probe and, interestingly, we found a high conductivity in the range of 1.8–2 × 10^6^ S/m, good resistivity that did not exceed 1–2 × 10^−6^ Ωm, and a sheet resistance lower than 16 Ω sq^−1^. The transparency values found using a spectrophotometer reached values beyond 85%, which indicates the high purity of the thin films. Atomic force microscopy indicated a smooth morphology with low roughness values for the films, indicating an adequate transitioning of the charges on the surface. Scanning electron microscopy was used to study the actual thicknesses and the morphology, through which we found no cracks or fractures, which implied excellent deposition and annealing. The X-ray diffraction microscopy results showed a high purity of the crystals, as the peaks (222), (400), (440), and (622) of the crystallographic plane reflections were dominant, which confirmed the existence of the faced-center cubic lattice of ITO. This work allowed us to design a method for producing excellent ITO thin films for solar-cell applications.

## 1. Introduction

Transparent conducting oxide (TCO) materials are widely used to transmit light through materials and conduct electricity. To produce these materials, metal oxides are used, which include tin oxide (SnO_2_), aluminum oxide (Al_2_O_3_), indium oxide (In_2_O_3_) [1], and zinc oxide (ZnO) [2,3]. There are many uses and applications of TCOs, such as display devices like flat-panel screens and touchscreens [4]; smart windows, in which the transparency can be controlled through applying a voltage and, hence, the heat and light transmitted through the window can be controlled [5]; and optoelectronic devices such as light-emitting diodes (LEDs) and photodetectors [6]. ITO films are of crucial importance in optoelectronic devices and solar cells. ITO is mainly used as the front electrode in photovoltaic applications [7] due to its high conductivity and transparency ranges, which make it a favorable choice for fabrication. Several studies have investigated the different properties of ITO and how they can be optimized using different growth techniques and conditions. In particular, in third-generation solar cells like perovskites and dye-sensitized solar cells, several attempts have been made to increase the efficiency of lab-built ITO to be used as a front electrode through different approaches. Although ITO has many optical, electrical, and physical advantages in the field of optoelectronic devices and photovoltaics, the high manufacturing costs and scarcity of indium and tin supplies hinder the long-term viability and scalability of its usage in third-generation solar cells.

The growth technique can contribute to material savings through reducing the amount of wasted material during the synthesis process. Radio frequency (RF) magnetron sputtering is a favored method for the growth of ITO films, as it provides high uniformity and precision when it comes to the microstructure growth and the ratios of indium and tin, which can be accurately specified for the sputtering targets. Unlike liquid and chemical approaches to creating ITO, which create a large amount of wasted materials, RF magnetron sputtering can create thousands of highly transparent and uniform thin films using one sputtering target. Many studies have focused on the creation of ITO thin films with RF magnetron sputtering. In a study by Vinh Ai Dao et al. [8], they heated the substrates in the deposition process and used an oxygen flow. Their results showed proper ITO parameters and, when they used a temperature of 200 °C, the heterojunction silicon solar-cell efficiency reached 16%. In another study by M.G. Sousa et al. [9], they compared their created ITO glass with and without the use of hydrogen pressure within the chamber and with only argon but an RF power of 250 W. This also resulted in adequate properties for the ITO to be used in solar-cell applications. In a study by F. Kurdesau et al. [10], they used oxygen in the chamber alongside argon. Another recent study by Amalraj P. A et al. [11] investigated the effects of the film thickness and RF power on the optical and electrical properties of ITO films. Shumin Y. et al. [12] prepared four different ITO targets in their lab with different crystallinities and studied the effects of the crystallinity of ITO targets on the properties of the ITO films. In the study of D. Kudryashov et al. [13], they found that, with argon gas, the best power at room temperature was only 50 W, and they tested a thickness of 100 nm with different parameters that resulted in the appropriate parameters for solar-cell applications. A study published after 2020 mostly tackled ITO growth within other layers (e.g., Cu) in RF magnetron sputtering [14]. Recent published studies focused on variations in power and growth temperature, using different gases or only argon gas (the latter was quite rarely used). For this reason, we studied indium tin oxide films grown using the radio frequency magnetron sputtering technique with varying thicknesses, in order to study the effect of thickness on the film’s properties using argon gas alone. Our approach had interesting results which contribute to the existing scientific literature.

## 2. Materials and Methods

### 2.1. Preparation of ITO Samples Using PVD

**Figure 1:**
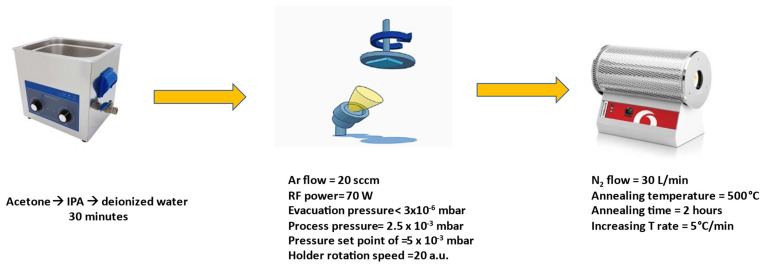
The fabrication process of ITO samples.

In this study, we used PVD (MiniLab ST060M R&D Magnetron Sputtering and Thermal Evaporation System, Moorfield Nanotechnology Limited. Cheshire, United Kingdom.) with load lock in the Environment and Sustainability Institute (ESI) solar lab to create films with different thicknesses using radio frequency sputtering. The ITO target was bought from Kurt J. Lesker, Sussex, United Kingdom, and had an indium to tin oxide ratio of 90/10 wt% In_2_O_3_/SnO_2_. The argon gas that was pumped to the chamber was of 99.99% purity and the silica low-iron glass substrates with a 4 mm thickness and 2 × 2 cm dimensions were obtained from Cornwall Glass Manufacturing, Plymouth, United Kingdom. The PVD was only run on the radio frequency magnetron sputtering system. The substrates were cleaned with acetone for 30 min, IPA for 30 min, and then deionized water for 30 min, all in an ultrasonic bath, and then dried in ambient air. The evacuation pressure of the chamber was less than 4 × 10^−3^ mbar. The ITO layers were manufactured in the radio frequency magnetron sputtering system chamber with a pressure set point of 5 × 10^−3^ mbar. The ITO sputtering during deposition was performed with an RF power of 70 W. The rotation speed for the substrate holder base was 20 a.u. and the Ar flow was 20 sccm. There was no increase in temperature on the base while growing the ITO films. Films with different thicknesses were grown: 50 nm, 100 nm, 150 nm, 200 nm, 250 nm, and 300 nm. As soon as the samples were manufactured, the samples were annealed at 500 °C for 2 h, with the temperature increasing at a rate of 5 °C per minute, in a tube furnace with a nitrogen gas flow (30 L/min) and they were left inside the furnace to cool to room temperature for another 2 h. Figure 1 illustrates the fabrication process with the different parameters used to grow the samples.

### 2.2. Characterization Techniques 

A four-point probe was used to study the films’ electrical properties, as our main purpose was to find the thickness that resulted in the best electrical characteristics to be used as a front electrode. After choosing the best thickness in the context of optimal electrical properties, the optical characterization of the samples before and after annealing was performed through measuring the spectral dependence of the transmission (T(k)) of deposited ITO layers within the visible wavelength range (300–800 nm) using a spectrophotometer; through this, we calculated the absorbance and found the bandgap. Moreover, the samples’ physical properties were examined using X-ray diffraction (XRD) (Bruker D8 advanced XRD, Bruker, Billerica, MA, USA); atomic force microscopy (AFM) (Bruker Innova AFM, Bruker, Billerica, MA, USA), after staining the samples with a thin carbon layer; and later, scanning electron microscopy (SEM) (Tescan Vega 3, Tescan, Brno, Kohoutovice Czech Republic). We have included the before and after annealing measurements in order to understand the influence of annealing on the optical and electrical properties.

## 3. Results and Discussion

### 3.1. X-ray Diffraction Microscopy Measurements

**Figure 2:**
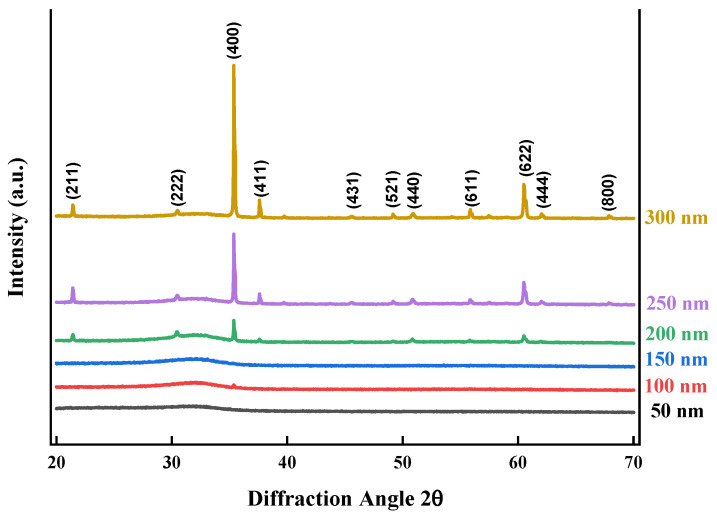
X-ray diffraction patterns of ITO films with different thicknesses before annealing.

Figure 2 shows the X-ray diffraction microscopy results for the films with different thicknesses before annealing. Almost all identifiable peaks lost some of their intensity, indicating that the ITO films had only a minimal amount of crystallinity before the annealing process. They had an amorphous quality for the most part. However, as the layer thickness increased, crystalline characteristics started to become more apparent.

**Figure 3:**
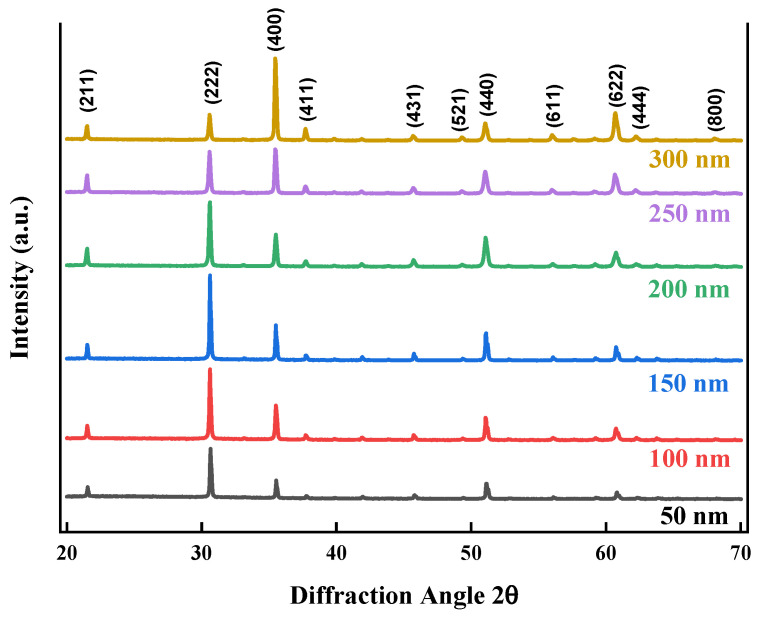
X-ray diffraction patterns of ITO films with different thicknesses after annealing.

Following annealing, the X-ray diffraction patterns (Figure 3) were characterized by prominent peaks that unmistakably signified an increase in crystallinity. Because charge carriers are given organized paths within the lattice structure of ITO, facilitating charge mobility, this phenomenon has implications for improving conductivity. The (222), (400), (440), and (622) peaks are the prominent peaks for the faced-center cubic lattice of ITO crystallographic plane reflections [15,16,17]. The other peaks ((211), (411), (431), (521), (611), (444), and (800)) represent other planes’ reflections. As the thickness increased, the preferential crystal orientation changed to the (400) plane, as the grain strain increased towards this plane due to interstitial oxygen and indium vacancies.

The formation of strain results from both external elements like dislocations and extended defects within the crystalline lattice, as well as intrinsic point defects like vacancies and site disorder. The calculated lattice constants showed a notable agreement with known reference values (JCPDS card No. 71-2194) [18,19,20].

### 3.2. Atomic Force Microscopy 

**Figure 4:**
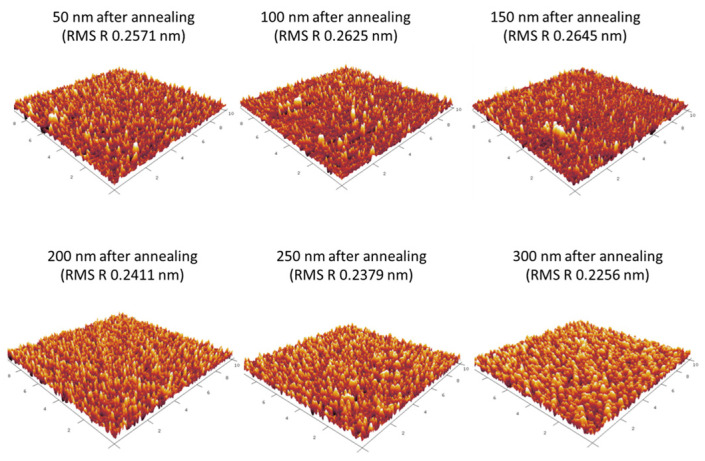
AFM images of ITO films with different thicknesses and their respective RMS R values.

Atomic force microscopy (AFM) measurements were used to gain an understanding of the surface morphology of the indium tin oxide (ITO) films. The experiment aimed to gain a thorough understanding of the complex interactions between the films’ electrical characteristics and their underlying physical characteristics. The goal was to identify the relationship between the surface morphology and the distinctive qualities of the ITO films. Figure 4 shows a variety of unique samples of ITO films with different thicknesses. The images all have dimensions of 10 µm × 10 µm. During the analysis of these images, an important observation became apparent. The mean value of the peaks and valleys determined over the total surface area was measured and recorded as the average roughness. This helped us to identify broad differences in the properties of the overall profile height. The square root of the distribution of surface height is known as the root mean square roughness (RMS R), which is thought to be more sensitive than the average roughness. It displays the profile heights’ standard deviation. In particular, each film had RMS R values that were consistently lower than the nominal criterion of 0.3 nm. This overall pattern was a reliable predictor of the crystallinity of the ITO material under the growth conditions of our experiment [10,21,22]. Similar RMS roughness results were found using magnetron sputtering (0.546 nm) [23] and another deposition technique (0.293 nm) [24]. Alternatively, we can see higher RMS roughness values found by Rita M. Carvalho et al. [25] and others [26,27,28] ranging between 3.9 nm up to 24.8 nm. We attribute these rough ITO surfaces to high temperatures used during growth and annealing as, in one method, they used thermal evaporation which can induce the formation of indium oxide on the surface, creating a clear variation; to the lack of annealing in proper and inert conditions; to the deposition parameters that included the distance between the target and the substrate; and to the gas pressure in the chamber and the sputtering power. Our low RMS roughness values can be attributed to the growth method in a vacuum which hindered the oxidation and formation of rough layers in inert conditions. Moreover, using an inert gas in the annealing method contributed to smoother films as indium tends to oxidize at high temperatures which can increase the formation of random indium oxide particles on the surface.

A recognizable pattern was revealed through further analysis of the RMS R values. In particular, values between 0.2571 nm and 0.2645 nm gradually emerged as the ITO layer’s thickness rose, covering the thickness range of 50 nm to 150 nm. However, after reaching a thickness of 200 nm, this pattern underwent a noticeable shift. The measured RMS R levels started to decrease at this point, going from 0.2411 nm to 0.2256 nm for thicknesses of 200 nm to 300 nm. We suggest that this phenomenon can be attributed to the natural transition taking place at the surface, which signaled the emergence of a more refined and smooth ITO film and an increase in grain sizes. The changes shown in Figure 4 resemble this perceptible growth, as the sharpness of the tip ends changed to a more curved layout while simultaneously showing an expansion in the grain dimensions. From our experiment, we can conclude that all the layers had optimal RMS R values and crystalline amorphous structures, which tended to have higher roughness and lower conductivity values. With increased roughness, we acquired a higher conductivity which was a result of the tips being closer to one another which helped in the transitioning of charges on the surface.

In summary, our utilization of atomic force microscopy in the analysis of the ITO films unveiled the intricate relationships between surface morphology, crystallinity, and electrical conductivity [29]. The low RMS R values signified the crystalline nature of the ITO films that were produced under our experimental conditions. The distinct trend of roughness value vs. thickness further solidifies this notion. 

### 3.3. Four-Point Probe

**Figure 5:**
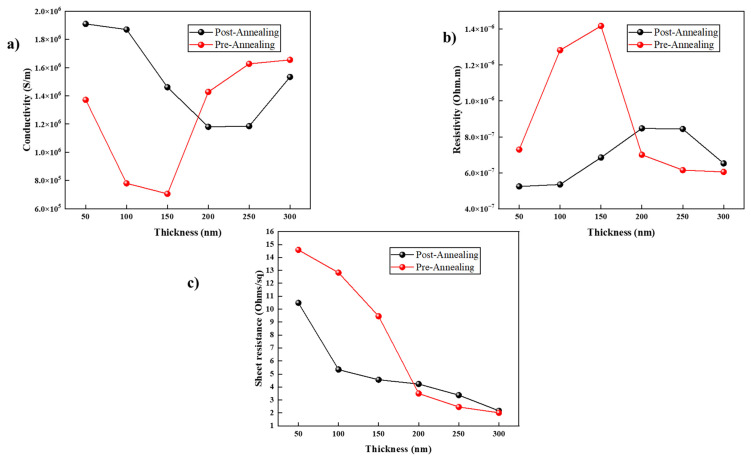
(**a**) Conductivity (S/m), (**b**) resistivity (Ωm), and (**c**) sheet resistance (Ω sq^−1^) vs. film thickness (nm).

Figure 5a–c show the electrical properties of the ITO films with different thicknesses before and after annealing. It is essential to understand the electrical properties to determine the quality of the deposited ITO [30].

Conductivity is one of the most important properties of an ITO thin film layer in third-generation solar-cell devices. In our films, we found that the conductivity values decreased with increasing thickness and then sharply increased after reaching 200 nm prior to annealing; this can be explained as thicker layers having higher conductivity due to reduced scattering sites and defects. However, thick layers tend to have poor crystallinity. On the other hand, after annealing, the films showed better conductivity values as the grain size increased; therefore, there was less boundary scattering [8], and the recrystallization reduced crystal defects and improved the electron mobility in the lattice [31] with better results at 50 nm and 100 nm (between 1.8–2 × 10^6^ S/m). It is important to consider that the optimal conductivity for ITO used in optoelectronic materials and solar cells should not be less than 1 × 10^4^ S/m [32]. Another important property for suitable ITO thin films in third-generation solar-cell devices is resistivity. Figure 5b illustrates the resistivity of the ITO samples with different thicknesses before and after annealing. Prior to annealing, the films had a high resistivity which was attributed to the low mobility of charge carriers. Nevertheless, the resistivity was quite stable post-annealing and was in the optimal range for solar-cell applications. A good resistivity for ITO for solar-cell applications is less than 1–2 × 10^−6^ Ωm [33,34]. In our results, we obtained values of 4 × 10^−7^ to 9 × 10^−7^ Ωm, indicating an optimal quality for the created films. It can be seen that the lowest and optimal resistivity was measured from the films with thicknesses of 50 nm and 100 nm. Figure 5c shows the pre- and post-annealing sheet resistance values of the samples. Annealing plays an important role in reducing the values of sheet resistance [35] which, in turn, is highly affected by the resistivity and the thickness of the layer. From the figure, we can observe that annealing improved the sheet resistance (R_sh_). Equation (1) links these variables together:

(1) $$R_{\mathrm{sh}}=\frac{\rho }{t}$$

where *ρ* is the resistivity of the thin film and *t* is the thickness of the thin film. It is expected that with increased thickness, we will have lower sheet resistance values. Nonetheless, the lower sheet resistance of these samples was also associated with a slightly lower conductivity and higher resistivity. Therefore, the optimal thickness based on the electrical properties was 100 nm. We could also opt for 50 nm but the film with this thickness showed a high sheet resistance that might affect the layers grown on the ITO and the efficiency of the solar device. We can conclude that annealing plays an important role in the improvement of ITO thin films [36].

### 3.4. Scanning Electron Microscopy

Scanning electron microscopy (SEM) was performed to confirm the actual thickness of each layer and to study the influence of the morphology on the optical, electrical, and mechanical properties of the films. 

**Figure 6:**
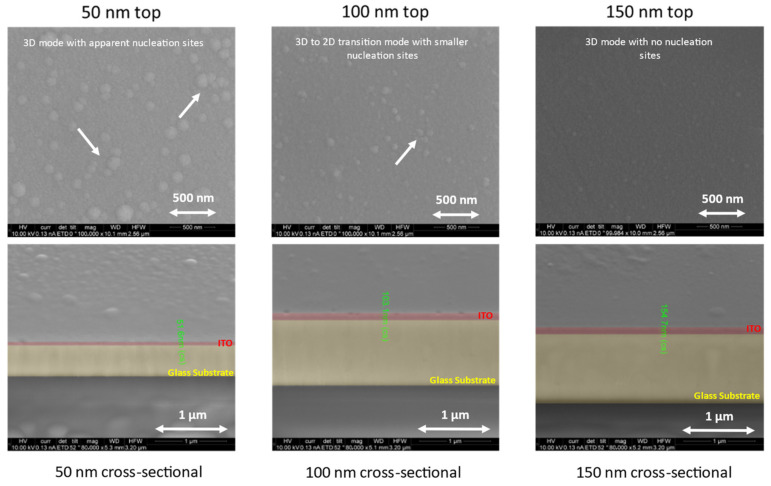
SEM images of top and cross-section of ITO films with thicknesses of 50, 100, and 150 nm after annealing.

We can see, from the images in Figure 6 and measurements, that the thickness of the films was accurate; this suggests high-precision growth using the radio frequency magnetron sputtering machine: the 50 nm film was measured as 51.6 nm, the 100 nm film was measured as 103.1 nm, and the 150 nm film was measured as 154.6 nm. The analysis revealed a shared characteristic among all the films: a uniform surface structure composed of nucleation sites pointed by white arrows in the figure that decreased in size as the thickness increased; this is because the Volmer–Weber island growth mode shifted to a Frank–van der Merwe mode, which is a transition from a 3D growth mode to a 2D growth mode, when the thickness was increased and the nodes and clusters were transformed into layers. This smoothness, which was free from both empty spaces and fractures, created good conductivity as fractures can contribute to extremely irregular electrical voltages on the surface [37,38]. 

### 3.5. Optical Properties 

**Figure 7:**
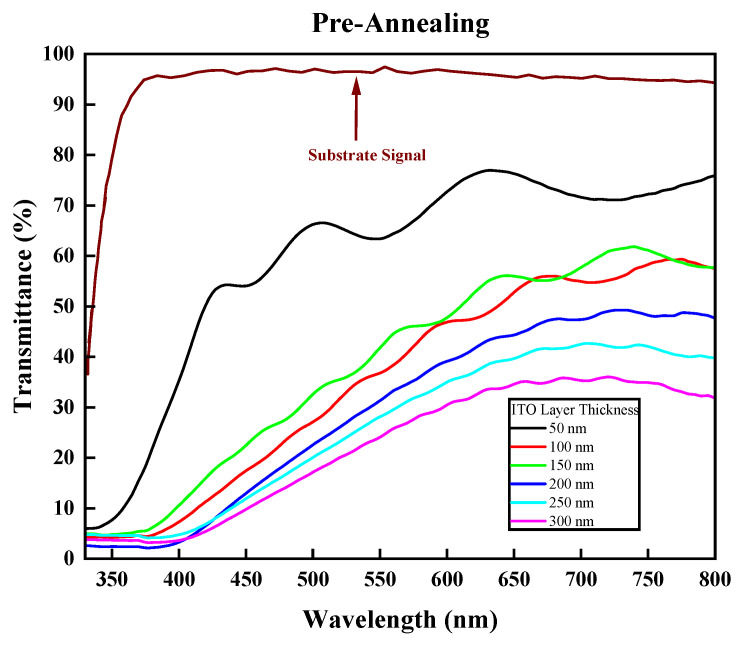
Transmittance (%) of films prior to annealing vs. wavelength (nm).

**Figure 8:**
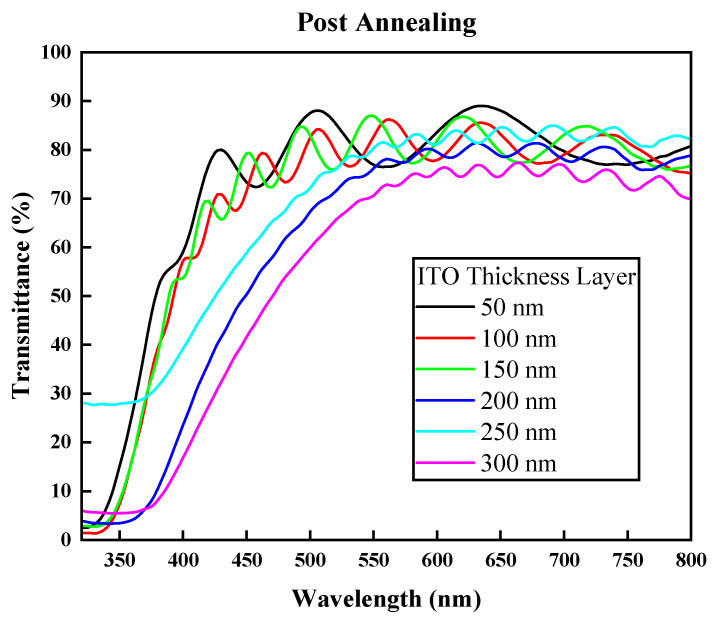
Transmittance (%) of films after annealing vs. wavelength (nm).

The final and most important property of ITO, which enables it to be installed or built into solar devices as the front electrode, is the transparency of the ITO film grown on the glass substrate. Initially, the silica low-iron glass substrate had a transmittance value of 94%. As can be seen in Figure 7, the transparency values prior to annealing were all below 85% [33,39,40], which are very poor transparency values due to the darker color of the ITO and the reflective properties it had before annealing. Furthermore, the post-annealing transparency (Figure 8) increased significantly in all samples with the best values observed in the films with thicknesses of 50 nm, 100 nm, and 150 nm with transparency values of 89%, 86.16%, and 86.92% at wavelengths of 635 nm, 560 nm, and 550 nm, respectively. This result indicates that these are highly transparent films that can be used for third-generation solar-cell applications [41]. Again, annealing the ITO samples was proven to improve the transmittance due to increasing the charge carrier numbers, with a better crystal quality and a decrease in grain boundary scattering as the grain sizes increased with temperature [42]. Although the 150 nm and 50 nm thicknesses showed better transparency values, they had a lower electrical quality compared to the 100 nm film.

Through our examination of the results, we found that the best thickness was 100 nm in terms of physical, electrical, and optical properties. Thus, we calculated the absorbance (A) of this sample using the transmittance data and Equation (2):

(2) $$A=2-log\left(T\%\right)$$

**Figure 9:**
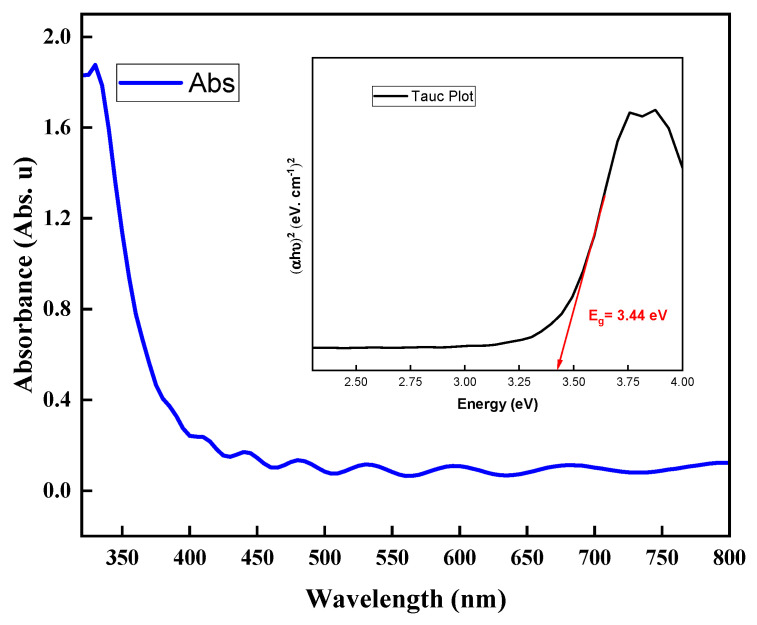
The absorbance of 100 nm ITO thin film with the calculated bandgap in the inset.

Figure 9 illustrates the resulting absorbance which was in the range of 0.1 to 0.2 Abs.u, indicating a highly favorable range [43,44], as the lowest absorbance possible is desirable for the application of electrodes in third-generation solar cells [7,45,46,47]. We used the absorbance to determine the bandgap using a Tauc plot, as shown in the inset of Figure 9. The resulting bandgap was 3.44 eV [48,49]. We validated the bandgap using the methods used by Jose C.S. Costa et al. [50] and Dariush Souri et al. [51] which indicated that the ITO film had highly transmittance and required the lowest energy from a photon to create voltage in any solar-cell circuit [52,53,54,55]. 

To provide more insights into our work, Table 1 summarizes the significant results of this study and compares them to those of previous studies.

## 4. Conclusions

In our recent research conducted at the ESI labs, we explored the growth of indium tin oxide (ITO) thin films using RF magnetron sputtering with argon gas. This approach is relevant for optoelectronic applications, particularly for third-generation solar cells. Through the various film thicknesses tested, we discovered that the films exhibited exceptional physical, electrical, and optical properties. However, the optimal thickness was identified as 100 nm. This particular thickness was distinguished by its remarkable transparency, exceeding 86%; low absorbance, ranging between 0.1 to 0.2 Abs.u; good bandgap energy of 3.44 eV coupled with superior electrical conductivity reaching about 1.86 × 10^6^ S/m; and low resistivity values of approximately 5.3 × 10^−7^ Ωm. These characteristics are crucial for ensuring the efficiency of optoelectronic devices used in the field of third-generation solar cells. Our SEM analysis confirmed that the surface morphology of these films was crack-free and validated the thicknesses achieved. Additionally, the ATM assessments indicated a uniform and gradual growth of the thin films with excellent RMS roughness values. Furthermore, the XRD analyses provided insights into the crystalline structure of the films, affirming their suitability for third-generation solar-cell applications. This research underscores the efficiency of RF magnetron sputtering in creating high-quality ITO films, with the 100 nm film particularly demonstrating promising properties for future technological innovations and competing with commercial ITO.

## Figures and Tables

**Table 1:** A comprehensive comparison between this work and previous studies.

Ref.	Year	Gas Type	Thickness (nm)	Annealing Temp (°C)	Electrical Properties	Transparency (%)	Band Gap (eV)	Morphological Properties
F. Kurdesau et al. [10]	2006	Argon–Oxygen	300–500	-	-	80–85	-	SEM: small-grained (10–20 nm) structure
Vinh Ai Dao et al. [8]	2010	Argon	100 ± 5	100	-	87–90	3.67–3.83	-
D Kudryashov et al. [13]	2013	Argon	~100	-	Resistivity: 5.4 × 10^−4^ Ω·cm	80–90	-	Smooth surface
A. P. Amalathas et al. [11]	2016	Argon	75–225	-	Average resistivity: 9.4 × 10^−4^ Ω·cm	Over 75	3.831–4.003	AFM: surface roughness increased with thickness
Shumin Yang et al. [12]	2020	Oxygen	~150	300	-	89.02–90.7	3.60–3.67	-
This work	2024	Argon	100	500	Resistivity range: 1–2 × 10^−4^ Ω·cm	86.16–89.0	3.44	Low RMS roughness values

## Data Availability

The data presented in this study are available upon request from the corresponding author.
